# Proton Therapy for Muscle-Invasive Bladder Cancer: Outcomes from the Multicenter Proton Collaborative Group PCG 001-09 Registry Trial

**DOI:** 10.7759/cureus.113689

**Published:** 2026-07-30

**Authors:** Caroline Oska, Keyur J Mehta, Charles B Simone, Kristin Hsieh, Madhur K Garg, Justin Tang, Arpit M Chhabra, J. Isabelle Choi, John H Chang, Arpi D Thukral Jr, Michael Durci, Elizabeth M Nichols, Ryan Grover, James Gray, Irini Yacoub

**Affiliations:** 1 Radiation Oncology, Montefiore Medical Center, Albert Einstein College of Medicine, Bronx, USA; 2 Radiation Oncology, New York Proton Center, New York, USA; 3 Radiation Oncology, Oklahoma Proton Center, Oklahoma City, USA; 4 Radiation Oncology, Northwestern Medicine Cancer Center and Proton Center, Warrenville, USA; 5 Radiation Oncology, Willis-Knighton Proton Therapy Center, Shreveport, USA; 6 Radiation Oncology, Maryland Proton Center Treatment, Baltimore, USA; 7 Radiation Oncology, Thompson Proton Center, Knoxville, USA; 8 Radiation Oncology, Tennessee Oncology Proton Center, Franklyn, USA

**Keywords:** bladder cancer, proton beam therapy (pbt), radiation, radiation and clinical oncology, trimodality therapy

## Abstract

Background: Muscle-invasive bladder cancer (MIBC) is treated with radical cystectomy or trimodality therapy (TMT), which yield comparable survival outcomes. Many patients undergoing TMT experience significant toxicity related to chemoradiation. The unique physics of proton radiation may allow for a more tolerable regimen. We herein report on the safety and efficacy of proton therapy for patients with MIBC.

Methods: Patients with non-metastatic MIBC treated with curative-intent proton radiation were enrolled in the multi-center Proton Collaborative Group (PCG) prospective, observational registry trial. Metastatic or palliatively treated patients were excluded. Baseline characteristics, including patient sex, age, stage, smoking history, and performance status, as well as details of the proton therapy, were documented. The primary endpoints were acute and late adverse events. Locoregional recurrence and overall survival were assessed.

Results: Between August 2010 and April 2025, 39 adult patients were eligible for analysis. The median age was 78 years (IQR 75-85), and most patients were White (36/39, 92.3%) and male (29/39, 74.4%). Most patients were clinical stage II (23/39, 59.0%). All patients received definitive proton therapy to a median (IQR) dose of 56.8 Gy (55.0-64.8), and 66.6% received concurrent chemotherapy. With a median follow-up of 11 months, there were three local recurrences and 14 deaths, yielding a crude 92.3% local control rate and 64.1% overall survival. The 12- and 24-month actuarial overall survivals using a Kaplan-Meier analysis were 59.0% and 32.7%, respectively. The highest grade of acute genitourinary or gastrointestinal toxicity was grade 3, affecting only 2 (5.1%) and 1 (2.6%) patients, respectively. No late grade 3 or higher toxicities were reported.

Conclusion: In the largest prospective cohort of MIBC patients treated with proton therapy reported to date, protons appear to be an effective and well-tolerated radiation modality for those undergoing TMT. This is increasingly important in the present landscape of expanding systemic therapies.

## Introduction

Muscle-invasive bladder cancer (MIBC) represents about 25% of all bladder cancer cases worldwide and is commonly treated with radical cystectomy and lymph node dissection [1]. This approach yields 5- and 10-year recurrence-free survival rates of around 80% and 70% in organ-confined cases (stage T2-T3a), respectively, and closer to 50-60% for more locally advanced disease (T3b-T4) [2]. However, for non-surgical candidates or patients who are recommended bladder preservation, trimodality therapy (TMT), consisting of maximal transurethral resection of the bladder tumor (TURBT) followed by chemoradiation, has evolved as an alternative [3,4]. Based on a pooled analysis of several RTOG studies, TMT yields 5- and 10-year disease-specific survival rates of 71% and 65%, respectively, with outcomes comparable to radical cystectomy in similarly staged patients [4].

The historical radiation technique used in prior studies for bladder preservation was 3D-conformal radiotherapy (CRT) using static fields to cover the target and multi-leaf collimators for blocking organs-at-risk [4,5]. This technique, however, is associated with significant morbidity. In the phase I-II RTOG study 99-06 evaluating TMT for bladder cancer, 26% of patients experienced acute grades 3 or 4 toxicity, 15% of which were gastrointestinal (GI) and another 4% were genitourinary (GU) [6]. Similarly, in RTOG 95-06, 21% of patients experienced grade 3 treatment-related bladder or GI toxicity, with another 18% suffering from grade 3 hematologic toxicity [7]. Apart from short-term side effects that may lead to treatment breaks and interruptions, the long-term toxicities of radiotherapy in this setting are also prevalent. An analysis of patients treated with TMT on RTOG 8903, 9506, 9706, and 9906 found a 10.2% late grade 2 pelvic toxicity rate, with late grade 3 toxicities reaching 7.0% [8]. The BC2001 trial compared whole-bladder radiation to reduced high-dose volume radiation therapy in an effort to minimize adverse events [9]. However, this did not result in a significant reduction in side effects, with 23% of all patients experiencing acute grades 3-4 toxicity and 13% reporting chronic grades 3-4 toxicity [9].

Intensity-modulated radiation therapy (IMRT) has gained interest in clinical trials and practice for its potential to alleviate radiation-induced toxicity in bladder cancer. IMRT, like 3DCRT, uses photon therapy, but it allows the radiation dose to better conform to the target's shape as the intensity of the radiation beams is controlled in multiple small volumes [10]. The phase II IMPART trial hypothesized that this technique would result in a more favorable toxicity profile for patients with node-positive bladder cancer [11]. They recruited 38 patients treated with bladder and pelvic nodal irradiation and found acute grade 3 GI and GU toxicities of 5.4 and 20.6%, respectively, and an overall late grade 3 toxicity rate of 5% [11]. The HIRACUM trial is actively recruiting MIBC patients to further investigate the side effects with a hypofractionated IMRT approach with concurrent chemotherapy.

Despite the ongoing search for more advanced techniques and modalities to improve bladder preservation outcomes, little attention has been given to proton therapy thus far. Through modulation of the Bragg-peak, proton therapy allows for significant sparing of normal tissues compared to 3D-CRT, IMRT, and volumetric modulated arc therapy (VMAT) across multiple other pelvic malignancies [12-14]. One prior systematic review on both retrospective and prospective data for proton therapy for MIBC found a three-year local control rate of 84.6% with only one grade 3 toxicity of urinary obstruction and no grade 3 GI toxicity [15].

These findings are encouraging and warrant further investigation on whether proton therapy can reduce toxicities in the era of organ preservation for bladder cancer. As the population of cancer survivors ages, mitigating short- and long-term toxicities of bladder cancer radiation is of utmost importance. Additionally, as chemo-immunotherapies continue to evolve, it becomes crucial that radiation plans optimally spare organs-at-risk that may otherwise be compromised by concurrent and adjuvant systemic treatments. We, therefore, analyzed a multi-institutional cohort of prospectively enrolled patients treated with proton therapy for non-metastatic bladder cancer. Our primary aim is to evaluate the safety profile of proton therapy for non-metastatic bladder cancer, as measured by the incidence and severity of acute and late adverse events. We secondarily report early locoregional control and overall survival outcomes. Given the limited existing prospective data on proton therapy in this disease site, we hypothesize that proton therapy is a safe and feasible radiation modality for patients undergoing bladder-preserving treatment, with acceptable rates of severe toxicity.

## Materials and methods

Study design and patient population

This is a multi-center, prospective, observational study of adult patients treated with a curative regimen of proton therapy for bladder cancer. Patients were enrolled in the Proton Collaborative Group (PCG) prospective registry trial, REG001-09. The PCG registry is a multi-institutional database that prospectively collects clinical, treatment, and outcomes data from patients receiving proton therapy across participating centers in the United States. Between August 2010 and April 2025, 68 consecutive patients with bladder cancer treated with proton radiotherapy were identified from the registry.

Given the limited existing data on proton therapy for bladder cancer, broad inclusion criteria were used to capture the heterogeneity of real-world clinical practice and treatment patterns. Patients were eligible for inclusion if they met all of the following criteria: (1) age ≥18 years at the time of treatment; (2) histologically confirmed bladder cancer; (3) treatment with proton radiotherapy delivered with curative intent; and (4) enrollment in the PCG REG001-09 registry trial. Patients were included regardless of the dose and fractionation of proton radiotherapy received. No restrictions were placed on prior therapies, including receipt of prior radiotherapy, prior TURBT history, or prior receipt of systemic therapy. Patients with regional lymph node involvement were also included. Patients were excluded if they had metastatic disease at the time of proton therapy or if the proton radiotherapy was delivered with palliative intent. These criteria resulted in 39 patients eligible for the final analysis.

Treatment planning and delivery

All patients underwent computed tomography (CT)-based simulation in the supine position using appropriate immobilization. Bladder filling/preparation and target delineation were performed according to institutional protocols. Target volumes were contoured based on available diagnostic imaging, cystoscopy findings, and contemporary consensus recommendations for bladder cancer radiotherapy. Clinical target volumes generally included the whole bladder, with elective pelvic nodal coverage incorporated per individual provider discretion. Daily cone beam CT scans were performed prior to each fraction, and verification CT scans were obtained per provider discretion on the need for adaptive replanning. Proton therapy was delivered using pencil beam scanning (PBS) or uniform scanning utilizing robust optimization for set-up and range uncertainties. Quality assurance was conducted according to institutional standards. Specific follow-up assessments, including clinical evaluation, cystoscopy, and imaging, were conducted per institutional protocols at each participating center. The PCG registry requests at least annual follow-up for as long as the enrolling sites are able to obtain it.

Data collection and endpoints

Clinical data were prospectively collected through the PCG registry, which extracts relevant information from participating institutions. Baseline characteristics were documented, including patient sex, age at diagnosis, clinical and/or pathologic stage, smoking history, and performance status, as well as any prior therapies received before proton therapy, including surgery, systemic therapy, and prior radiotherapy. Details of the radiation therapy regimen, including the dose, timing, and targets, were noted, along with the receipt of concurrent and sequential systemic therapy.

The primary endpoints were the incidence and severity of acute and late adverse events according to the Common Terminology Criteria for Adverse Events (CTCAE) version 5.0 [16]. Acute toxicities were defined as those with onset within 90 days of the start of radiotherapy, whereas late toxicities were defined as those with onset beyond 90 days. Secondary endpoints included locoregional recurrence, defined as any cancer recurrence in the bladder or regional lymph nodes, and overall survival, defined as the time from the first day of radiotherapy to death from any cause or last known follow-up. The follow-up period was defined as the time interval between the first day of radiotherapy and the patient’s last known contact in the electronic medical record.

Statistical analysis

Descriptive statistics were used to summarize baseline patient and treatment characteristics. Continuous variables were reported as medians with interquartile ranges (or means with standard deviations, as appropriate), and categorical variables were reported as frequencies and percentages. Overall survival was estimated using the Kaplan-Meier method, with actuarial survival rates calculated at 12 and 24 months. Patients who were alive at last follow-up were censored at the date of last known contact. All statistical analyses were performed using IBM SPSS Statistics for Windows, Version 29.0 (Released 2022; IBM Corp., Armonk, New York).

## Results

Between the years 2010 and 2025, 39 adult patients with non-metastatic bladder cancer who completed a curative regimen of proton radiotherapy were enrolled. Baseline patient characteristics are summarized in Table 1. Patients were predominantly White (36/39, 92.3%), non-Hispanic (30/39, 76.9%), and male (29/39, 74.4%). The median age was 78 years (IQR 75-85). Most patients had a good performance status, with 69.2% having an Eastern Cooperative Oncology Group (ECOG) score of 0 or 1 [17]. Over half of the population had a smoking history (23/39, 59.0%), with three patients reporting active smoking at the time of radiotherapy.

**Table 1:** Baseline patient characteristics.

Baseline characteristics	N (%)
Gender	
Male	29 (74.4)
Female	10 (25.6)
Age in years, Median (IQR)	78 (75-85)
Race	
White	36 (92.3)
Other	1 (2.6)
Not reported	2 (5.1)
Ethnicity	
Hispanic	1 (2.6)
Non-Hispanic	30 (76.9)
Not reported	8 (20.5)
ECOG	
0	12 (30.8)
1	15 (38.5)
2	4 (10.3)
3	1 (2.6)
Not reported	7 (17.9)
Smoking history	
Yes	23 (59.0)
No	12 (30.8)
Not reported	4 (10.3)
Clinical Group Stage	
I	1 (2.6)
II	23 (59.0)
III	11 (28.2)
Not reported	4 (10.3)
Clinical T stage	
T1	2 (5.1)
T2	24 (61.5)
T3	6 (15.4)
T4	2 (5.1)
Not reported	5 (12.8)
Clinical N stage	
N0	30 (76.9)
N1	2 (5.1)
N2	1 (2.6)
Not reported	6 (15.4)
Histology	
Urothelial cell	26 (66.7)
Papillary	3 (7.7)
Squamous cell	2 (5.1)
Adenocarcinoma	2 (5.1)
Small cell	1 (2.6)
Not reported	5 (12.8)
Grade	
1	1 (2.6)
2	2 (5.1)
3	5 (12.8)
4	3 (7.7)
Not reported	28 (71.8)
Treated for recurrence	5 (12.8)

Thirty-five of the 39 patients had staging information documented in the database, and this was according to the AJCC 8th edition for bladder cancer. Of the 35 patients with available TNM data, the majority were clinical stage II (23/35, 65.7%), followed by clinical stage III (11/35, 31.4%). The most common clinical T stage was T2, affecting 24 of 34 (70.6%) evaluable patients, and most patients did not have lymph node involvement (30 of 33 evaluable patients, 90.9%). Urothelial carcinoma was the most common histology, affecting 26 of the 39 patients (66.7%), followed by papillary (3/39, 7.7%), and then squamous cell (2/39, 5.2%). Most patients (71.8%), unfortunately, did not have a grade specified in the database, although there were five patients with documented grade 3 disease and another 3 with grade 4 disease.

Among relevant prior cancer-directed treatment history, five patients were treated for non-metastatic bladder cancer recurrence, whereas the remainder were treated for a primary occurrence of disease. Three patients had also undergone a prior course of pelvic radiation, all of which were for prostate cancer: IMRT to the prostate only, LDR brachytherapy to the prostate, and proton therapy to the prostate and seminal vesicles. Prior to receiving definitive proton radiotherapy for their bladder cancer, seven (17.9%) received prior chemotherapy, and another two (5.1%) were previously treated with intra-vesicular Bacillus Calmette-Guérin (BCG). Additionally, prior to undergoing their definitive proton radiotherapy, at least 25 of 39 (64.1%) had documentation of prior TURBT, and only two were reported to have positive margins. As TURBT is a near-universal component of TMT, it is likely the true proportion of those who received one was higher, though explicit documentation in the database was limited.

Definitive treatment characteristics are summarized in Table 2. Twenty-six (66.6%) patients had documentation of concurrent chemotherapy with their proton radiation. The most commonly used chemotherapy agents were cisplatin (received by 11 of the 26 patients, 42.3%) and gemcitabine (received by 8 of the 26 patients, 30.8%). Per the study’s methods, all 39 patients in this cohort underwent a definitive course of proton radiotherapy. More than half of these (21/39, 53.8%) were treated to the bladder only, whereas the remainder (18/39, 46.2%) were treated to the bladder and pelvic lymph nodes. The median (IQR) radiation dose received was 56.83 Gy (55.0-64.8), and the median (IQR) number of fractions received was 30 (21-33.5). The bladder-only median (IQR) radiation dose was 56.28 Gy (55-64.8). During proton radiotherapy, only two patients experienced treatment interruptions that were due to toxicity.

**Table 2:** Treatment characteristics.

Treatment characteristics	
Concurrent chemotherapy, N (%)	26 (66.7)
Chemotherapy type, N (%)	
Cisplatin	7 (26.9)
Cisplatin/Gemcitabine	3 (11.5)
Gemcitabine	4 (15.4)
Gemcitabine/Carboplatin	1 (3.8)
Carboplatin	1 (3.8)
Carboplatin/Etoposide	1 (3.8)
5-fluorouracil/Mitomycin C	3 (11.5)
Capecitabine	1 (3.8)
Cisplatin/Paclitaxel	1 (3.8)
Paclitaxel	1 (3.8)
Other	3 (11.5)
Radiation, N (%)	
Bladder only	21 (53.8)
Bladder with lymph nodes	18 (46.2)
Median (IQR) radiation dose	56.83 Gy (55.0-64.8)
Median (IQR) radiation fraction number	30 (21-33.5)
Median (IQR) radiation dose to bladder only	56.28 (55-64.8)

The median follow-up was 11 months. During follow-up, there were three local recurrences, which yielded a crude local control rate of 92.3%. Five patients suffered from distant metastatic disease without concurrent local recurrences. Sites of distant recurrences included the lungs, bowel, and bones. Fourteen patients died during the follow-up period, of which six were attributed to bladder cancer. This yields a crude overall survival rate of 64.1%. The 12- and 24-month actuarial overall survival rates using a Kaplan-Meier survival analysis were 59.0% and 32.7%, respectively.

Results for adverse events (according to CTCAE v5.0) are summarized in Table 3. There were nine patients who reported one or more symptoms at baseline (prior to receiving radiation). The most frequently reported baseline symptoms were fatigue (N = 5), urinary frequency (N = 5), urinary urgency (N = 4), and anorexia (N = 3). Twenty-four total patients reported one or more acute adverse events. The most common acute adverse event was fatigue (N = 15), most (12/15) of which were grade 1. Regarding acute urinary symptoms, there were five patients (12.8%) with grade 2 urinary adverse events: urinary frequency (N = 2), incontinence (N = 1), urgency (N = 1), and non-infective cystitis (N = 1). Grade 3 urinary toxicity affected only two patients (5.1%): urinary tract pain and a kidney infection, which was likely unrelated to treatment. No patients experienced an acute grades 4 or 5 adverse urinary event. Regarding acute GI symptoms, there were only two patients who experienced grade 2 adverse events: nausea and dysgeusia. One patient (2.6%) experienced acute grade 3 proctitis. There were no grade 4 or higher acute GI events. There was one grade 3 event of hypocellular bone marrow in a patient who had received concurrent carboplatin/etoposide, as well as additional chemotherapy courses prior to the definitive chemo-radiation course. Only two patients experienced a treatment interruption related to acute toxicity. Regarding late toxicity, there were no grade 3 or higher adverse events affecting any organ system, including GU and GI. Four grade 2 GU and GI late adverse events were noted--these included one each of urinary frequency, urinary incontinence, urinary urgency, and proctitis. None of these were in patients who received prior radiation, and all of them had a smoking history.

**Table 3:** Baseline, acute, and late adverse events (CTCAE v5.0).

Adverse event	Baseline symptom		Acute toxicity		Late toxicity	
	Grade	N (%)	Grade	N (%)	Grade	N (%)
Urinary						
Dysuria	1	1 (2.6)	1	1 (2.6)	-	-
Hematuria	1	2 (5.1)	1	3 (7.7)	-	-
Frequency	1	4 (10.3)	1	7 (17.9)	1	2 (5.1)
	2	1 (2.6)	2	2 (5.1)	2	1 (2.6)
Incontinence	1	2 (5.1)	1	3 (7.7)	1	1 (2.6)
			2	1 (2.6)	2	1 (2.6)
Retention	1	2 (5.1)	1	4 (10.3)	1	1 (2.6)
Urgency	1	3 (7.7)	1	8 (20.5)	2	1 (2.6)
	2	1 (2.6)	2	1 (2.6)	-	-
Urinary tract pain	1	1 (2.6)	1	5 (12.8)	-	-
	2	1 (2.6)	3	1 (2.6)	-	-
Non-infective cystitis	-	-	1	1 (2.6)	-	-
Kidney infection	-	-	3	1 (2.6)	-	-
Gastrointestinal						
Diarrhea	1	1 (2.6)	1	2 (5.1)	1	1 (2.6)
Fecal incontinence	1	1 (2.6)	-	-	-	-
Rectal hemorrhage	1	1 (2.6)	-	-	-	-
Nausea	1	1 (2.6)	1	7 (17.9)	-	-
	-	-	2	1 (2.6)	-	-
Vomiting	-	-	1	3 (7.7)	-	-
Abdominal Pain	-	-	1	1 (2.6)	-	-
Constipation	-	-	1	3 (7.7)	-	-
Dysgeusia	-	-	2	1 (2.6)	-	-
Dysphagia	-	-			1	1 (2.6)
Proctitis	-	-	3	1 (2.6)	2	1 (2.6)
Vaginal discharge	-	-	1	1 (2.6)	-	-
Anorexia	1	3 (7.7)	1	3 (7.7)	-	-
Chills	1	1 (2.6)	-	-	-	-
Dyspnea	1	1 (2.6)	-	-	-	-
Erectile dysfunction	1	1 (2.6)	2	1 (2.6)		
Esophagitis	1	1 (2.6)	-	-	-	-
Fatigue	1	5 (12.8)	1	12 (30.8)	1	1 (2.6)
	-	-	2	3 (7.7)	-	-
Hyperlipidemia	1	1 (2.6)	-	-	-	-
Hypertension	1	1 (2.6)	-	-	-	-
Weight loss	1	1 (2.6)	1	1 (2.6)	-	-
Pain	1	1 (2.6)	-	-	-	-
Pelvic pain	-	-	1	1 (2.6)	-	-
	-	-	2	2 (5.1)	-	-
Back pain	-	-	-		1	1 (2.6)
Bone marrow hypocellular	-	-	3	1 (2.6)	-	-
Dehydration	-	-	2	1 (2.6)	-	-
Dermatitis	-	-	1	2 (5.1)	-	-
Dizziness	-	-	1	1 (2.6)	-	-
Fever	-	-	1	1 (2.6)	-	-
Headache	-	-	1	1 (2.6)	-	-
Peripheral neuropathy	-	-	-	-	2	1 (2.6)

## Discussion

The purpose of the present study is to report early clinical efficacy and safety of proton radiotherapy for the treatment of non-metastatic bladder cancer using data from a large, multi-institutional prospective registry. Our results demonstrate a mild toxicity profile with definitive proton therapy across a broad range of clinical contexts and treatment protocols, with only three patients who experienced any acute grade 3 or higher GI or GU adverse event, despite 46.2% receiving pelvic nodal irradiation with a resulting larger volume of abdominopelvic organs-at-risk irradiated. Additionally, one of the grade 3 GU toxicities was a kidney infection, which was likely unrelated to proton radiotherapy, as this patient did not exhibit any obstructive urinary toxicities, such as retention, during or after treatment. Only two patients experienced a treatment interruption related to toxicity. Moreover, no patient experienced any grade 3 or higher late toxicities, which is relevant in this aging patient population.

The toxicity rates observed in our cohort are contextualized alongside published data from photon-based series; however, heterogeneity in patient selection, treatment protocols, staging, follow-up duration, and study design preclude direct comparison. Among photon-based techniques, IMRT specifically intends to provide better target conformality while protecting surrounding normal structures compared to 3D-CRT. It utilizes a complex computerized inverse-planning system to adjust many treatment variables with greater freedom [18,19]. Despite these dosimetric advantages, the IMPART trial reported an acute grade 3 GI and GU toxicity rate of 26%, over three times that in the present cohort, and a late grade 3 toxicity rate of 5%, whereas no grade 3 late toxicities were observed in our study [11]. These findings are despite only 47% of patients in the IMPART population receiving concurrent chemotherapy compared to 67% in the present population. However, the IMPART study treated both the bladder and pelvic nodes as their population was either node-positive or high-risk node-negative, whereas only around half of the patients in our population received nodal irradiation, which, along with the shorter follow-up, may account for some of the discrepancy. Another study by Turgeon et al. evaluated IMRT for elderly (age 70+ years old) patients with bladder cancer undergoing TMT with curative intent. They found only a 4% acute grade 3 GI or GU toxicity rate, but up to 17% of patients experienced acute grade 3 or 4 hematologic and/or liver toxicities, indicating difficulty with a combined-modality approach in this population [20]. Another study by Sherry et al. compared IMRT to 3D-CRT for bladder cancer and found a 21% grade 3 GU toxicity rate and an overall 5% grade 3 GI toxicity rate, although not all patients received pelvic nodal irradiation [21]. The accompanying dosimetric data from that study confirmed a significantly lower V40, V45, V50, V55, and V60 with IMRT vs 3D-CRT (p < 0.0001) for the rectum and bowel, as well as a lower volumetric dose to the bladder (p < 0.01) [21]. More recently, the international phase II RAIDER trial randomized patients to whole bladder radiation, standard-dose adaptive radiotherapy, or dose-escalated adaptive radiotherapy using IMRT. They found only up to a 1.7% grade 3 or higher toxicity rate with adaptive dose-escalation, indicating that adaptive planning may further improve upon these IMRT toxicity outcomes [22].

While IMRT significantly reduces the toxicities of bladder radiation compared to 3D-CRT, it still leaves around 20% of patients facing severe adverse events. The unique physics of protons, including their low entrance dose, linear travel path, characteristic Bragg peak, and lack of exit dose, may allow for lower rates of toxicities without compromising tumor coverage [23,24]. These physical properties introduce planning considerations unique to proton therapy. Proton dose distributions are highly sensitive to changes in tissue density and organ position along the beam path, making consistent bladder filling--typically an empty bladder protocol per NCCN guidelines [25]--and careful beam angle selection to avoid traversing mobile bowel or air-filled structures essential for plan robustness. Unlike photon IMRT, which relies on geometric PTV margin expansion, proton planning employs robust optimization that incorporates setup and range uncertainties into the optimization process [26]. Daily volumetric imaging with cone beam CT is standard to verify positioning and assess anatomical changes that may warrant adaptive replanning. Although patient-level setup and dosimetric data were not available from our registry, these planning strategies are typically adopted in institutional standards. In doing so, various organ sites have been able to demonstrate a dosimetric, and sometimes clinical, advantage with proton therapy. For example, in rectal cancer, protons offer reduced volumetric doses to structures such as the small bowel, bladder, and bone marrow, compared to both photon-based IMRT and 3D-CRT [12]. In endometrial cancer patients who require extended field radiotherapy, proton therapy has been shown to significantly reduce bone marrow and bowel doses compared to photon-based IMRT, resulting in less grade 3+ hematologic toxicity [14]. More relevant to the present study is a prospective trial by Araya et al. evaluating proton therapy in 36 patients with MIBC [15]. Patients received 40-41.4 Gy in 20-23 fractions to the pelvic cavity or entire bladder, followed by a boost of 19.8-36.3 Gy in 10-14 fractions to the tumor site(s) given with concurrent chemotherapy. While achieving overall survival, progression free survival, and local control rates of 90.8, 71.4, and 84.6%, respectively, there was only one case (2.8%) of grade 3 GU toxicity, and no severe GI toxicity was observed [15]. In a systematic review, these toxicity rates were compared to those who got photon-based therapy, which conversely experienced weighted mean grade 3 or higher GI and GU toxicity rates of 6.2 and 2.2%, respectively [15].

While sparing normal tissues with protons may not directly impact the efficacy of bladder radiation, it may certainly improve the tolerability of TMT. For instance, our study only had one patient (2.6%) with a grade 3 non-GI or GU adverse event (hypocellular bone marrow), compared to the 17% found in the study by Turgeon et al. [20]. Although not specifically assessed in this study, the ability to spare bone marrow and other organ function in our population with proton therapy is invaluable, especially in the setting of concurrent systemic agents that are also bone marrow suppressive and/or have toxic effects on multiple organs. Inspired by the CheckMate 274 trial that supports adjuvant nivolumab as a standard of care option for MIBC after radical resection [27], other studies aim to investigate the addition of further chemo-immunotherapies to TMT. For example, NEXT is a phase II trial that assessed adjuvant nivolumab following chemoradiotherapy in patients with MIBC, and they found a 10.7% rate of grade 3 adverse events [28]. Another new trial is actively recruiting entitled, “Phase Ib/II Study of Enfortumab Vedotin and Pembrolizumab Combined with Radiotherapy as a Bladder-Sparing Trimodality Therapy in Muscle Invasive Bladder Cancer” [29]. Enfortumab Vedotin is an antibody-drug conjugate, which may raise concerns regarding its safety in coincidence with radiotherapy. A meta-analysis demonstrated that concurrent antibody drug conjugates significantly increase the risk of symptomatic radionecrosis following radiosurgery for patients with metastatic breast cancer [30]. While one retrospective review abstract suggested no increase in safety events for patients receiving antibody drug conjugates with radiation therapy for urothelial carcinoma [31], more robust prospective data are necessary. As we await these and future trial results that incorporate novel agents to TMT, proactively optimizing the toxicity profile of existing ones is essential. Proton radiotherapy is one such modality that may improve the feasibility and tolerability of combined modality therapy.

Apart from the low rates of severe toxicity, patients in our cohort achieved a high locoregional control rate of 92.3% over 11 months. In BC2001, the two-year locoregional control rate with 3D-CRT was 61-64%, and this came at the expense of 23% of the population experiencing an acute grade 3-4 toxicity [9]. The long-term results of the pooled RTOG meta-analysis on TMT demonstrated a 43% local failure rate at five years, of which 30.2% were muscle-invasive [4]. Compared to IMRT, our early results appear similar, though longer follow-up in a more standardized, comparative protocol is certainly needed. The locoregional control rate in the study by Turgeon et al. was 91.7% at a median 28-month follow-up, and that of the study by Sherry et al. was 79% at a median follow-up of 10.3 months [20,21]. Our 92.3% locoregional control rate exists in the setting of nearly 40% of the cohort not reported to have received a TURBT, and an unclear proportion of which were maximal resections.

At the time of last follow-up, 64% of our patients remained alive. Our actuarial 12-month overall survival was 59.0%. The RTOG meta-analysis showed a five-year overall survival of 57% with TMT [4], while BC2001 had five-year survival rates of between 38% and 44% [9]. In keeping with the more modern techniques of studies utilizing IMRT, Turgeon et al. found an overall survival rate of 61% at a median of 28 months, while Sherry et al. reported overall survival rates between 60 and 70% at their 10.3-month median follow-up. While our actuarial overall survival rates were fairly low, only 6 of the 14 deaths in our cohort were noted to be related to the bladder cancer. Our median follow-up is also shorter than those in these historical trials. One study found the median time to recurrence of bladder cancer after radical cystectomy to be around 13 months [32]. Another study found the median time to recurrence to be 27 months after radical treatment (which may have included post-operative radiation therapy) [33]. As such, any concrete conclusions of local control or overall survival in our population are significantly limited by our 11-month median follow-up. However, our early toxicity data, which depends less on long-term follow-up, still provides preliminary support for a tolerable safety profile of bladder proton therapy.

Limitations of this study include firstly its non-randomized nature, which inherently introduces selection bias even within a prospective registry design. Additionally, although all patients were treated with curative regimens for non-metastatic disease, there was heterogeneity in the radiation dose received, the radiation targets (bladder vs bladder and lymph nodes), and the remainder of the documented TMT regimens employed. For example, while TMT involves maximal TURBT followed by concurrent chemoradiation, the registry did not consistently report this data; our true rates of TURBT and receipt of chemotherapy were therefore likely higher than herein reported. The variability in our data is largely due to the registry-based design, which captures real-world practice patterns across multiple institutions rather than enforcing a standardized protocol. Similarly, complete clinical staging information was not available for all patients, and some did not have a tumor grade specified, which limits the ability to fully characterize the cohort and may affect the generalizability of our findings. Another limitation includes our relatively short follow-up, which limits the ability to draw durable conclusions regarding local control and overall survival. An updated report on this cohort will be of interest in the future. Lastly, the actuarial 12- and 24-month survival rates were somewhat lower than anticipated. Less than half of deaths were attributed to bladder cancer, which is supported by the overall good local control rates. However, given the nature of the registry, additional potential confounders could not be extracted to provide a potential explanation, and our overall small sample size limits our statistical power in survival analyses and subgroup comparisons. We also acknowledge that patients selected for proton therapy may represent a favorably selected population, as access to proton therapy centers and insurance approval may introduce socioeconomic and geographic selection bias. Despite these limitations, prospective registry data can provide value in the setting of relatively rare clinical scenarios, and, at the time of our analysis, bladder cancer patients represented only 3.9% of the overall PCG registry population.

## Conclusions

In conclusion, in the largest reported cohort of prospectively enrolled patients with bladder cancer treated with proton therapy to date, proton therapy is safe and feasible for patients undergoing TMT, achieving overall low rates of severe acute and late toxicities. These findings are hypothesis-generating and warrant validation in larger, prospective comparative studies. Given the present evolving treatment landscape that may include concurrent chemotherapy with or without immunotherapies, limiting treatment toxicity while maintaining efficacy is increasingly critical. Proton therapy warrants further study in bladder cancer to permit more widespread use in centers with the available resources.

## References

[1] Jubber I, Ong S, Bukavina L (2023). Epidemiology of bladder cancer in 2023: a systematic review of risk factors. Eur Urol.

[2] Stein JP, Lieskovsky G, Cote R (2001). Radical cystectomy in the treatment of invasive bladder cancer: long-term results in 1,054 patients. J Clin Oncol.

[3] Shipley WU, Kaufman DS, Zehr E (2002). Selective bladder preservation by combined modality protocol treatment: long-term outcomes of 190 patients with invasive bladder cancer. Urology.

[4] (2015). Errata. J Clin Oncol.

[5] Shipley WU, Winter KA, Kaufman DS (1998). Phase III trial of neoadjuvant chemotherapy in patients with invasive bladder cancer treated with selective bladder preservation by combined radiation therapy and chemotherapy: initial results of Radiation Therapy Oncology Group 89-03. J Clin Oncol.

[6] Kaufman DS, Winter KA, Shipley WU (2009). Phase I-II RTOG study (99-06) of patients with muscle-invasive bladder cancer undergoing transurethral surgery, paclitaxel, cisplatin, and twice-daily radiotherapy followed by selective bladder preservation or radical cystectomy and adjuvant chemotherapy. Urology.

[7] Kaufman DS, Winter KA, Shipley WU (2000). The initial results in muscle-invading bladder cancer of RTOG 95-06: phase I/II trial of transurethral surgery plus radiation therapy with concurrent cisplatin and 5-fluorouracil followed by selective bladder preservation or cystectomy depending on the initial response. Oncologist.

[8] Efstathiou JA, Bae K, Shipley WU, Kaufman DS, Hagan MP, Heney NM, Sandler HM (2009). Late pelvic toxicity after bladder-sparing therapy in patients with invasive bladder cancer: RTOG 89-03, 95-06, 97-06, 99-06. J Clin Oncol.

[9] Huddart RA, Hall E, Hussain SA (2013). Randomized noninferiority trial of reduced high-dose volume versus standard volume radiation therapy for muscle-invasive bladder cancer: results of the BC2001 trial (CRUK/01/004). Int J Radiat Oncol Biol Phys.

[10] Bakiu E, Telhaj E, Kozma E, Ruçi F, Malkaj P (2013). Comparison of 3D CRT and IMRT tratment plans. Acta Inform Med.

[11] Tan MP, Harris V, Warren-Oseni K (2020). The Intensity-Modulated Pelvic Node and Bladder Radiotherapy (IMPART) Trial: A phase II single-centre prospective study. Clin Oncol (R Coll Radiol).

[12] Colaco RJ, Nichols RC, Huh S (2014). Protons offer reduced bone marrow, small bowel, and urinary bladder exposure for patients receiving neoadjuvant radiotherapy for resectable rectal cancer. J Gastrointest Oncol.

[13] Fok M, Toh S, Easow J, Fowler H, Clifford R, Parsons J, Vimalachandran D (2021). Proton beam therapy in rectal cancer: a systematic review and meta-analysis. Surg Oncol.

[14] Xu MJ, Maity A, Vogel J, Kirk M, Zhai H, Both S, Lin LL (2018). Proton therapy reduces normal tissue dose in extended-field pelvic radiation for endometrial cancer. Int J Part Ther.

[15] Araya M, Ishikawa H, Nishioka K (2023). Proton beam therapy for muscle-invasive bladder cancer: a systematic review and analysis with Proton-Net, a multicenter prospective patient registry database. J Radiat Res.

[16] (2017). National Cancer Institute. Common Terminology Criteria for Adverse Events (CTCAE) Version 5.0. Bethesda, MD: U.S. Department of Health and Human Services, National Institutes of Health; Published November 27. https://dctd.cancer.gov/research/ctep-trials/for-sites/adverse-events/ctcae-v5-5x7.pdf.

[17] Oken MM, Creech RH, Tormey DC (1982). Toxicity and response criteria of the Eastern Cooperative Oncology Group. Am J Clin Oncol.

[18] Pirzkall A, Carol M, Lohr F, Höss A, Wannenmacher M, Debus J (20001). Comparison of intensity-modulated radiotherapy with conventional conformal radiotherapy for complex-shaped tumors. Int J Radiat Oncol Biol Phys.

[19] Cheung K (2006). Intensity modulated radiotherapy: advantages, limitations and future developments. Biomed Imaging Interv J.

[20] Turgeon GA, Souhami L, Cury FL, Faria SL, Duclos M, Sturgeon J, Kassouf W (2014). Hypofractionated intensity modulated radiation therapy in combined modality treatment for bladder preservation in elderly patients with invasive bladder cancer. Int J Radiat Oncol Biol Phys.

[21] Sherry AD, Stewart A, Luo G, Kirschner AN (2019). Intensity-modulated radiotherapy is superior to three-dimensional conformal radiotherapy in the trimodality management of muscle-invasive bladder cancer with daily cone beam computed tomography optimization. J Radiat Oncol.

[22] Huddart R, Hafeez S, Griffin C (2025). Dose-escalated adaptive radiotherapy for bladder cancer: results of the phase 2 RAIDER randomised controlled trial. Eur Urol.

[23] Liu H, Chang JY (2011). Proton therapy in clinical practice. Chin J Cancer.

[24] Dicello JF (2007). Absorption characteristics of protons and photons in tissue. Technol Cancer Res Treat.

[25] (2026). NCCN Bladder Cancer Guidelines (Version 2.2026, published June 22, 2026). https://www.nccn.org/professionals/physician_gls/pdf/bladder.pdf.

[26] Unkelbach J, Paganetti H (2018). Robust proton treatment planning: physical and biological optimization. Semin Radiat Oncol.

[27] Bajorin DF, Witjes JA, Gschwend JE (2021). Adjuvant nivolumab versus placebo in muscle-invasive urothelial carcinoma. N Engl J Med.

[28] Galarza Fortuna GM, Grass D, Maughan BL (2025). Nivolumab adjuvant to chemo-radiation in localized muscle-invasive urothelial cancer: primary analysis of a multicenter, single-arm, phase II, investigator-initiated trial (NEXT). J Immunother Cancer.

[29] Koshkin Koshkin, V V (2017). Phase Ib/II study of enfortumab vedotin and pembrolizumab combined with radiotherapy as a bladder-sparing trimodality therapy in muscle invasive bladder cancer. ClinicalTrials.gov identifier NCT06470282. Published June 19. NCT06470282.

[30] Koide Y, Kodaira T (2024). Concurrent antibody-drug conjugates and radiotherapy: a new perspective on radiation necrosis in HER2-positive breast cancer brain metastases from the DESTINY-Breast03 and HER2CLIMB trials. ESMO Open.

[31] Lock D, Soni YS, Hwang L (2026). Safety of combining radiation therapy and antibody drug conjugates in advanced urothelial and other cancers. Adv Radiat Oncol.

[32] Mitra AP, Quinn DI, Dorff TB (2012). Factors influencing post-recurrence survival in bladder cancer following radical cystectomy. BJU Int.

[33] Anghel RM, Gales LN, Trifanescu OG (2016). Outcome of urinary bladder cancer after combined therapies. J Med Life.

